# Lateral Flow Loop-Mediated Isothermal Amplification Test with Stem Primers: Detection of* Cryptosporidium* Species in Kenyan Children Presenting with Diarrhea

**DOI:** 10.1155/2018/7659730

**Published:** 2018-02-26

**Authors:** Timothy S. Mamba, Cecilia K. Mbae, Johnson Kinyua, Erastus Mulinge, Gitonga Nkanata Mburugu, Zablon K. Njiru

**Affiliations:** ^1^Department of Biochemistry, Jomo Kenyatta University of Agriculture and Technology, P.O. Box 62000-0200, Nairobi, Kenya; ^2^Centre for Microbiology Research, Kenya Medical Research Institute, P.O. Box 19464-00202, Nairobi, Kenya; ^3^School of Health Sciences, Meru University of Science and Technology, P.O. Box 972-60200, Meru, Kenya; ^4^School of Health Professions, Murdoch University, Mandurah Campus, Education Drive, Mandurah, WA 6210, Australia

## Abstract

*Background. Cryptosporidium* is a protozoan parasite and a major cause of diarrhea in children and immunocompromised patients. Current diagnostic methods for cryptosporidiosis such as microscopy have low sensitivity while techniques such as PCR indicate higher sensitivity levels but are seldom used in developing countries due to their associated cost. A loop-mediated isothermal amplification (LAMP) technique, a method with shorter time to result and with equal or higher sensitivity compared to PCR, has been developed and applied in the detection of* Cryptosporidium* species. The test has a detection limit of 10 pg/*µ*l (~100 oocysts/ml) indicating a need for more sensitive diagnostic tools. This study developed a more sensitive lateral flow dipstick (LFD) LAMP test based on SAM-1 gene and with the addition of a second set of reaction accelerating primers (stem primers).* Results*. The stem LFD LAMP test showed analytical sensitivity of 10 oocysts/ml compared to 100 oocysts/ml (10 pg/ul) for each of the SAM-1 LAMP test and nested PCR. The stem LFD LAMP and nested PCR detected 29/39 and 25/39 positive samples of previously identified* C. parvum* and* C. hominis* DNA, respectively. The SAM-1 LAMP detected 27/39. On detection of* Cryptosporidium* DNA in 67 clinical samples, the stem LFD LAMP detected 16 samples and SAM-2 LAMP 14 and nested PCR identified 11. Preheating the templates increased detection by stem LFD LAMP to 19 samples. Time to results from master mix preparation step took ~80 minutes. The test was specific, and no cross-amplification was recorded with nontarget DNA.* Conclusion.* The developed stem LFD LAMP test is an appropriate method for the detection of* C. hominis, C. parvum,* and* C. meleagridis *DNA in human stool samples. It can be used in algorithm with other diagnostic tests and may offer promise as an effective diagnostic tool in the control of cryptosporidiosis.

## 1. Background

Cryptosporidiosis is caused by a group of phenotypically, and genotypically diverse* Cryptosporidium *species [1] and transmission of infection occurs when an individual comes in contact with infective oocyst(s) via contaminated food, water, person-to-person contact [2–4], and contact with animals. Transmission is common in developing countries due to poor sanitation and limited access to safe drinking water. The disease affects enterocytes of the small intestines and is a major cause of diarrhea, hospitalization, malnutrition in children [5–7] and can be fatal among immune-compromised persons [3, 4]. The ability of low doses of oocysts to cause infection following exposure and the absence of sensitive and effective diagnostic tools and treatment regime make cryptosporidiosis a major public health concern [5, 8].

In Sub-Saharan Africa and South Asia where most diarrheal disease deaths occur, there is limited data on the burden of* Cryptosporidium* diarrheal cases [9]. Nevertheless, the prevalence of the infection is thought to range from 10 to 33% among Human Immunodeficiency Virus (HIV) infected adult persons [10] and can even be higher in children. Indeed, findings from an Ethiopian based study indicated the presence of* Cryptosporidium* species in 26.9% of HIV-positive patients [11], while a prevalence of 73.6% was recorded among HIV-positive children in Uganda presenting with persistent diarrhea [12]. Kenyan based studies involving children have revealed a higher cryptosporidiosis prevalence of up to 30.5% among HIV-infected participants compared to noninfected participants in the same cohort [5, 13]. The enormous* Cryptosporidium* disease burden among immune-compromised children warrants development of sensitive diagnostics tools among other control tools.

Routine diagnosis of cryptosporidiosis in Kenya and in most of the developing world is based on microscopic detection of* Cryptosporidium *oocysts in stool samples despite the method's low sensitivity and inability to distinguish between pathogenic and nonpathogenic* Cryptosporidium* species [14]. There are over 20 species and numerous genotypes of* Cryptosporidium* of which* C. hominis* and* C. parvum* are the most common species infecting humans [9, 15]. Several tests have been developed to detect pathogenic strains and among them are ELISA and immune chromatography [16]. Others include pathogen DNA-based tests such as real-time PCR [17], single strand conformation polymorphism [18], and restriction fragment length polymorphism [19]. PCR tests have indicated superior sensitivity and specificity compared to antigen-based tests. However, the method has limited use in the routine diagnosis of cryptosporidiosis in Kenya due to its associated costs.

In the last decade, loop-mediated isothermal amplification (LAMP) has emerged as a powerful technique for the diagnosis of parasites of both medical and veterinary importance [20]. The method is robust and can amplify DNA from partially processed samples [21]. It has a shorter time to results compared to PCR, and the large amounts of reaction products formed allow the use of various detection formats [22, 23] making it ideal for application under field conditions. LAMP has been used in the diagnosis of protozoan parasitic pathogens such as* Plasmodium falciparum *[24], human infective* Trypanosoma rhodesiense* [21] and pathogens found in stool such as* Ascaris lumbricoides* [25] and* Necator americanus* [26]. Various methods have been used to detect LAMP products [27] in particular; the use of lateral flow dipstick (LFD) method offers high test specificity and visual inspection of results. This is because the detection probe targets a specific complementary sequence in the product and the results appear as presence or absence of a line on LFD stick [28, 29]. This makes LFD ideal for field application. Previously, LAMP tests for* Cryptosporidium* species have been designed and evaluated based on the heat shock protein 70 (HSP 70) gene, glycoprotein 60 (GP60), and the S-adenosyl-methionine synthetase-1 (SAM-1) gene [1, 15]. Among the three tests, the SAM-1 LAMP test indicated a higher sensitivity level by detecting 10 pg/*μ*l compared to 1 ng/*μ*l for the LAMP targeting GP60 gene [15]. These detection levels are inadequate for deployment of LAMP tests for routine diagnosis of cryptosporidiosis. Therefore, considering the advantages of LAMP and its potential as a diagnostic tool in rural endemic setups, there is a need to explore ways of improving the diagnostic capacity of cryptosporidiosis LAMP tests.

Recent studies have indicated that it is possible to improve the detection limits of some LAMP tests through the addition of a second set of reaction accelerating primers (stem primers) that target the stem section of the LAMP amplicon [30]. The advantage of stem primers is that they can be used in addition to loop primers without affecting the LAMP test reproducibility [30]. Here we report an improved LAMP test for the detection of human infective* Cryptosporidium *species that uses stem primers and detection of the LAMP products using a lateral flow dipstick format.

## 2. Methods

### 2.1. Clinical Samples and DNA Extraction

This study used a total of 39 archived DNA samples determined as* C. parvum* and* C. hominis* through sequencing of* Cryptosporidium* GP60* gene* [6]. The DNA were extracted from stool samples obtained from children aged 5 years and below presenting with diarrhea at the pediatric ward of Mbagathi district hospital and from three participating clinics (Lea Toto, Medical Missionaries of Mary, and Reuben Center) within Mukuru slums, Nairobi County [5]. To evaluate the developed LAMP test, 67 DNA samples were prepared from stool samples collected from children who presented with diarrhea in the same study area. The stool samples were preserved in 2.5% potassium dichromate and stored at −80°C. Genomic DNA was processed from the preserved specimen using the QiAmp® DNA Stool Mini Kit (Qiagen, West Sussex, United Kingdom) as per the manufacturer instructions with slight modifications. Briefly, 200 *μ*l of fecal suspension was washed five times with triple-distilled water by centrifugation at 14,000 rpm for 5 minutes per cycle. To this suspension 1.4 ml of ASL buffer was added and subjected to five times' thawing at 80°C and freezing at −80°C to rupture the oocysts. A 100 *μ*l suspension was used for DNA extraction, and the resulting genomic DNA was then eluted in 50 *μ*l of nuclease-free water and stored at −20°C until use.

### 2.2. Design of LAMP Primers and Probe

The sequence section of SAM-1 gene for three common species of* Cryptosporidia *affecting humans in Kenya* C. parvum* (AB119646.1),* C. hominis* (X662396.1), and* C. meleagridis* (AB119648.1) were obtained from Genbank. The gene target was selected based on the findings of a recent study that indicated that LAMP test developed based on the SAM-1 gene achieved the best sensitivity levels [15]. The sequences were aligned using Clustal Omega program (http://www.ebi.ac.uk/Tools/msa/clustalo/) and the most conserved sequence section determined and used to design LAMP primers. Briefly, the forward and backward outer primers (F3/B3) and the forward and backward inner primers (FIP/BIP) were designed using the primer explorer version 3 software (https://primerexplorer.jp/e/). The loop forward and backward primers (LF/LB), stem forward and backward primers (SF/SB), and the probe were designed manually following published conditions [29, 30]. All the primers were checked for target specificity using the nucleotide basic local alignment search tool (BLASTn) https://blast.ncbi.nlm.nih.gov/Blast.cgi.

### 2.3. Optimization of LAMP Reactions

The designed LAMP primers were first analyzed for sensitivity using a 10-fold serial dilution of* C. hominis *and* C. parvum* reference DNA initially prepared from 10^7^ oocysts suspended in 10 ml of fecal materials and using standard LAMP conditions [32] to select the most sensitive primer set (Table 1). This was followed by the optimization of reaction components (reagents) using the Taguchi method to select the optimum concentration of the four reagents determined to have the greatest effect on LAMP reaction. Briefly, the reaction components concentration was varied at three levels and ranged within 30–60 pmoles for inner primers, 10–30 pmoles for loop primers, 10–30 pmoles for stem primers, and 1–3 mM for dNTPs. The concentrations variables were then arranged in an orthogonal array and used to determine the amount of LAMP product formed [33]. This was followed by regression analysis to determine the concentration optima for each selected reaction component [33]. To select the optimal reaction temperature, LAMP reactions were performed at 61, 63, and 65°C, respectively. The tests were run for 30–60 minutes to obtain products. The LAMP products were analyzed using electrophoresis in 2% agarose gel.

### 2.4. Lateral Flow LAMP Reactions

The selected LAMP primers (Table 1) and determined reaction concentrations were used with the forward inner primer being labeled with biotin in the 5′-end and the probe for detecting biotinylated LAMP product labeled with fluorescein isothiocyanate (FITC). Briefly, the Taguchi method determined the concentrations for inner primers at 44 pmoles, with both stem and loop primers 20 pmoles and dNTPs at 2 mM. The concentration of other reagents was as reported previously [20]. The template was 2 *μ*l for both* C. hominis* and* C. parvum* DNA. The reactions were done at 63°C for 60 minutes followed by reaction inactivation at 80°C for 5 minutes. After the LAMP reaction, the LFD hybridization was performed by incubating LAMP products with 20 pmol of FITC-labeled probe at 63°C for 5 min in a final volume of 20 *μ*l followed by the addition of 8 *μ*l of the reaction mixture and 150 *μ*l of the reaction assay buffer. The LFD strips (Millennia® HybriDetect, Millennia Biotec, Germany) were then dipped into the mixture for 5 min at room temperature. The test was considered positive when both the control and test lines appeared. The size of the obtained amplicon was 220 bp. The experimental test was labeled stem LFD-LAMP. The reactions were duplicated using an opened heating block that maintained the temperature at ~62-63°C.

### 2.5. Stem LFD-LAMP and Nested PCR Analytical Sensitivity and Analysis of Clinical Samples

The analytical sensitivity of the stem LFD-LAMP test was determined using tenfold serial dilution of* C. hominis* and* C. parvum* DNA. A sequenced* C. parvum* and* C. hominis* DNA [6] were used as positive controls. The* C. meleagridis* DNA was not used in sensitivity analysis because the concentration was very low. Two formats of stem LFD-LAMP tests (i.e., with outer F3/B3 primers and without outer primers) were compared with the SAM-1 LAMP test [15] and nested PCR targeting* Cryptosporidium* species small subunit rRNA [31] (Table 2). All clinical samples were analyzed in duplicate and repeated once after two weeks. To check whether the stem LFD-LAMP format analytical sensitivity could be improved further, preheated templates were used. Briefly, the LAMP master mix was divided into 25 *μ*l reaction tubes and placed in the incubation chamber at ~63°C. After approximately 3 minutes, 2 *μ*l of preheated template (genomic DNA) was added to each respective tube and reactions were left to run for 60 minutes. To check time to results for different LAMP formats, a dilution of 10^−4^ (~1000 oocysts/ml) of reference* C. hominis* DNA was used, and reactions ran for 25, 30, 35, 40, and 45 minutes. For each time schedule, the reaction tubes were transferred to a thermal block set at 80°C to stop the reaction. For SAM-1 LAMP and nested PCR, the expected products were analyzed using 2% agarose gel. The stem LFD-LAMP test specificity was checked using* Toxoplasma gondii, Giardia duodenalis, Entamoeba histolytica*,* Ascaris lumbricoides, Cyclospora *species, and human DNA.

### 2.6. Detection and Confirmation of Stem LAMP and Nested PCR Product

The formation of LAMP product was first monitored through gel electrophoresis in 2.0% agarose gel and through addition of 1/10 dilution of SYBR® Green 1 dye. Later the product detection was exclusively done using the lateral flow dipstick format. Two approaches were used to confirm that the LAMP test amplified the predicted product, namely, the restriction enzyme digestion and sequencing. The restriction enzyme* Nde*l (New England BioLabs, MA, USA) which has a single cut site within the selected product sequence was used to digest LAMP product at 37°C for 3 h, followed by electrophoresis in 3% agarose gel. The predicated two bands were sized with molecular markers. Secondly, the uppermost LAMP amplicon were excised from the agarose gel, cloned, and transformed and inserts sequenced using an automated DNA 3730 analyzer. The resulting sequences were aligned with the target sequences using the DNAman computer software (Lynnon, USA). In nested PCR, the two-step restriction digestion of the secondary PCR products was carried out using endonucleases* SspI* and* VspI* and* Cryptosporidium* species and genotypes determined analyzed as described previously [34].

## 3. Results

### 3.1. Detection and Confirmation of LAMP Products

**Figure 1:**
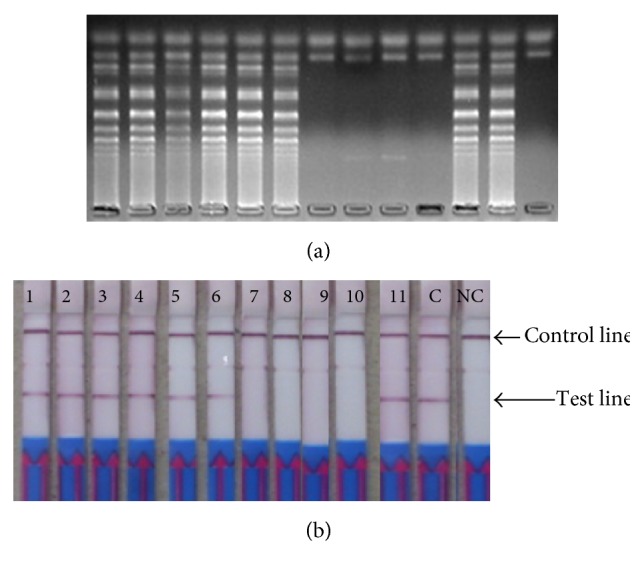
The detection* Cryptosporidium *spp. using the stem LAMP amplification product (220 bp) using 2.0% agarose gel stained with ethidium bromide and LFD format. The genomic DNA was prepared from specimen collected from children presenting with diarrhea. The faint line between the test line and the positive control line is nonspecific binding at DIG test line because the strips were done to detect two products. 1 = MB407, 2 = MB419, 3 = MB491, 4 = MB501, 5 = MB502, 6 = M1492, 7 = M1599, 8 = M009, 9 = M016, 10 = M044, 11 = M074, C =* C. hominis* DNA, and NC = PCR water.

**Figure 2:**
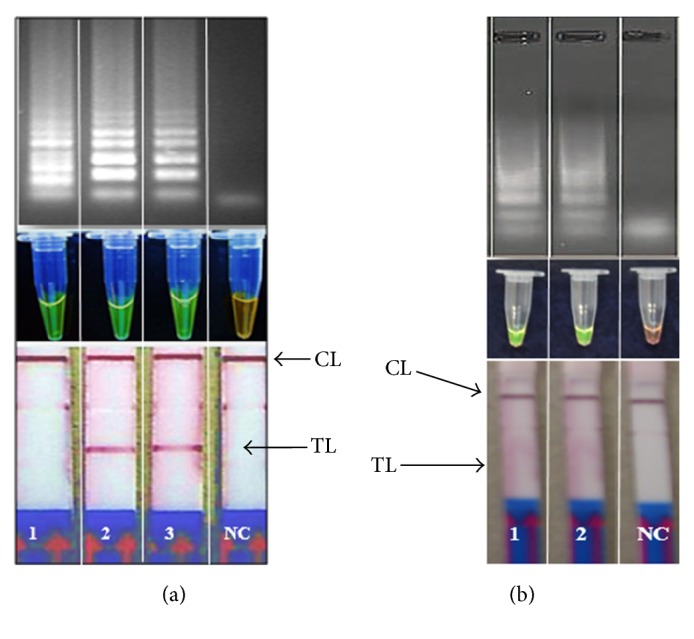
The detection of stem LAMP product from some selected reactions done for over 60 minutes using 2.0% agarose gels stained with ethidium bromide, SYBR Green 1 dye, and LFD dipstick format. 1 = (false positive), 2 =* C. hominis*, 3 =* C. parvum, *and NC = PCR water. (b) The appearance of nonspecific products at 75 minutes' reaction cut-off time. 1, M044 (false positive), 2, M099 (false positive), and NC = PCR water. TL = test line; CL = control line. The nonspecific products show different patterns agarose gel and turn green on addition of SYBR Green 1. However, none was positive using the LFD format.

**Figure 3:**
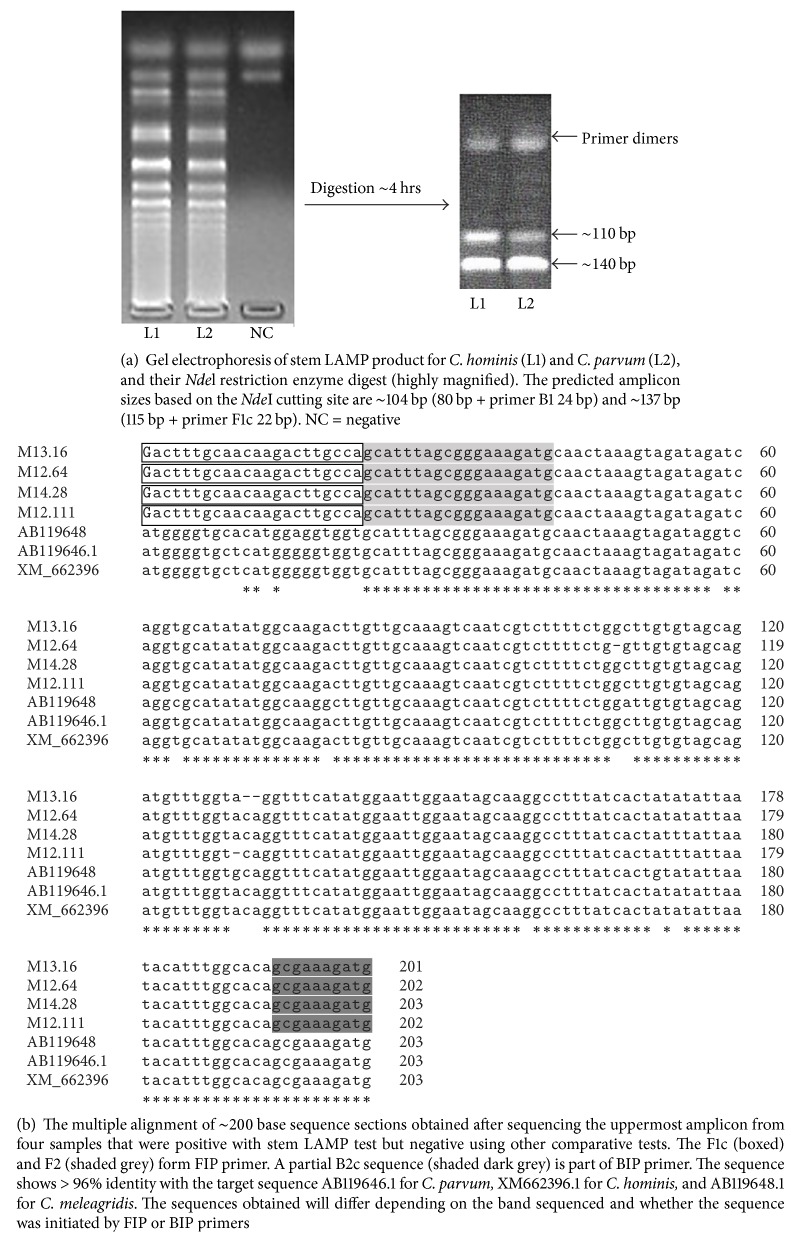


The positive stem LAMP products showed ladder-like pattern on the agarose gel indicating the formation of stem-loops with inverted repeats (Figure 1(a)) and the expected test line on the LFD strip (Figure 1(b)). Occasional nonspecific products were noted in the agarose gels if the LAMP reactions were run for over 70 minutes and which turned green (false positive) on the addition of SYBR Green 1 dye (Figures 2(a) and 2(b)). These bands varied in patterns from reaction to reaction while consistent patterns were recorded for true positive samples (Figure 1(a)). The false positive samples were not detectable with the LFD strips (Figures 2(a) and 2(b)). The restriction enzyme digestion of the stem LAMP amplicons indicated the predicted amplicons of approximately 117 bp and 103 bp, respectively (Figure 3(a)). The sequence from the uppermost amplicon of randomly selected stem LAMP positive samples indicated high sequence homology with SAM gene sequences from* C. hominis* and* C. parvum* (Figure 3(b)). No restriction enzyme digestion was recorded from samples with inconsistent banding patterns. The LAMP test was reproducible using the open heat block, and no cross-reactivity was recorded with nontarget DNA from* Toxoplasma gondii, Giardia duodenalis, Entamoeba histolytica*,* Ascaris lumbricoides, Cyclospora *spp*., *or human DNA.

### 3.2. Analytical Sensitivity of LAMP and Nested PCR Tests

The stem LFD-LAMP indicated unequivocal detection limit of 10 oocysts/ml using the 10-fold serial dilution of the* C. hominis* and* C. parvum* reference DNA. However, an average of 2 of 6 replicates in every run, or approximately 30% of the replicates consistently showed detection limit of ~1 oocyst/ml when the template was preheated (Tables 2 and 3). The SAM-1 LAMP test and the nested PCR indicated detection limit of 100 oocysts/ml (Table 2). Time to results from master mix preparation was 80 minutes for the stem LFD-LAMP format and 120 minutes for SAM-1 LAMP test using gel electrophoresis (Table 3).

### 3.3. Clinical Samples Result

The stem LFD-LAMP and nested PCR detected 29/39 and 25/39 positive samples of previously identified* C. parvum* and* C. hominis* DNA, respectively. The SAM-1 LAMP detected 27/39 (Table 3). On detection of* Cryptosporidium* DNA in 67 clinical samples, the stem LFD-LAMP detected 16 samples and SAM-2 LAMP 14 and nested PCR identifies 11. Preheating the templates increased detection by stem LFD-LAMP to 19 samples.

## 4. Discussion

Loop-mediated isothermal amplification of DNA is a method that has gained momentum in the diagnosis of different microorganisms due to its inherent advantages of high sensitivity and specificity and its potential applicability in resource-poor endemic areas [20]. In this study, we successfully used a second set of reaction accelerating primers (stem primers) combined with a lateral flow dipstick format to design a sensitive LAMP test capable of detecting* C. hominis, C. parvum,* and* C. meleagridis* based on the SAM-1 gene. The stem LFD-LAMP test indicated a shorter time to results, higher analytical sensitivity, and better comparative analysis, a characteristic that translated to superior detection of pathogen DNA in clinical specimen (Table 3) when compared to SAM-1 LAMP and nested PCR. The higher detection levels of stem LFD-LAMP test may be attributed to the use of two reaction accelerating primers (loop and stem primers) which lead to formation of larger amounts of product compared to the SAM-1 LAMP format which relies only on loop primers. The loop primers accelerate the reaction by priming the sequence loops [35] while the stem primers accelerate the reaction by targeting the stem section of the sequence [30]. The use of preheated template marginally improved the stem LFD-LAMP test sensitivity by 10-fold and detection of pathogen DNA by ~4.6% from clinical specimen compared to SAM-1 LAMP test (Tables 2 and 3). It can be assumed that preheating of the template unwinds target DNA and hence provides more target for priming. Moreover, heating accelerates betaine destabilization of the target DNA bonds, hence easier displacement by the outer primers. The omission of outer F3 and B3 primers in this stem LAMP format indicated poor test performance (Table 2) confirming that outer primers have varied effects on different stem LAMP tests [30]. The higher detection of rates of* Cryptosporidium* DNA from clinical samples compared to SSU rRNA nested PCR agrees with previous results [15, 36].

There was a general agreement in the detection of the stem LAMP products using gel electrophoresis and LFD (Figures 1(a) and 1(b)). However, SYBR Green 1 dye could not differentiate some false positive products limiting its use as detection format in this assay. This is because intercalating dyes bind to any double stranded DNA including the primer dimers (Figures 2(a) and 2(b)) leading to erroneous results interpretation. The appearance of false positive products was further confirmed by failure of digestion using restriction enzyme* Nde*l compared to the positive products that gave the predicted amplicons of 117 bp and 103 bp, respectively (Figure 3(a)). Theoretically, LAMP test should not amplify nonspecific products since amplification specificity is supposedly enhanced by using several primers. Nevertheless, spurious products are formed if the test is not optimized or left to run for too long (Figures 2(a) and 2(b)). Determination of nonspecific products is valuable since their presence reduces the amplification efficiency and ultimately the accuracy of the test. Since most LAMP product detection formats are developed for visual inspection of results, a product confirmation step ought to be built into the test development protocol and/or a specific detection product format is recommended [29]. In this regard, the designed LFD format in this study showed superior specificity to the intercalating dyes. The dipstick format relies on a specific DNA sequence probe that binds to a specific complementary sequence in LAMP product. The lateral flow strips used in this study have dual detection ability for FITC- and DIG-labeled products, but only the FITC was used (Figure 1, Table 2). There is a nonspecific faint line at DIG section (Figure 1) that does not affect the results interpretation. Nonetheless, it indicates the need of using specific FITC-labeled strips only.

All samples that were positive with nested PCR and SAM-1 LAMP test were also positive with stem LFD-LAMP test (Table 3) indicating that the tests were detecting the same thing. Moreover, specificity of the stem LAMP test was confirmed through sequencing of product from four samples (Figure 3(a)). The stem LFD-LAMP assay described here can further be improved by using a dipstick cartridge which allows insertion of the sample followed by a locking mechanism that cuts and pours the product directly into the LFD strip. The development of such technologies will eliminate the need to open the tube and potentially reduces contamination. Since stem LFD-LAMP test is faster to perform, the technique could form part of diagnostic algorithms for* Cryptosporidium* species detection where it can be used to select cases for further analysis.

## 5. Conclusion

This work reports the use of stem primers and lateral flow dipstick format to improve the detection of* Cryptosporidium* oocyst DNA from stool samples. The LFD format showed superior specificity in detection of the target DNA compared to DNA intercalating dyes and without compromising the test sensitivity. To advance the LFD format, a novel single-step reaction that will allow direct detection of product with the LFD strips without necessarily opening the tube needs to be considered. Such integration of key technologies will contribute towards making stem LFD-LAMP a suitable complementary test to the current tests (microscopy and PCR) used in the detection of cryptosporidiosis, especially in resource-poor countries.

## Figures and Tables

**Table 1:** Nucleotide sequences for *Cryptosporidium hominis* and *parvum *for stem SAM-1 LAMP test. Degenerate primers have been underlined and made bold.

Target	Primer name	Sequence (5′-3′)	Bases	Final amplicon size
SAM-1	F3	GAGGATGGGGTGCTCATGG	19	220 bp
	B3	CCTTATTAACTATCTCCAG**Y**AG	22	
	FIP	GACTTTGCAACAAG**Y**CTTGCCAGCATTTAGCGGGAAAGATG	41	
	BIP	ATTGGAATAGCAA**R**GCCTTTATCGTCATTATACCCATCTTTCGC	44	
	LF	C**R**CCTGA**Y**CTATCTACTTTAG	21	
	LB	CT**R**TATATTAATACATTTGGCAC	23	
	SF	TACACAA**K**CCAGAAAAGACG	20	
	SB	TGTTTGGT**R**CAGGTTTCATATG	20	
	Probe	CTTGTGTAGCAGATGTTTGGTACAGG	26	

**Table 2:** The analytical sensitivity of stem LFD LAMP test formats, SAM-1 LAMP, and PCR tests using a 10-fold serial dilution of *C. hominis *DNA.

Test	Primer combination	Probe	10-fold serial dilution						Result (Min)^e^	Remarks
			10^−1 to 3^	10^−4d^	10^−5^	10^−6^	10^−7^	10^−8^		
Stem LFD LAMP^a^	F3/B3, FIP/BIP, LF/LB, SF/SB	FITC	+	+	+	+	±^†^	−	*30*	This study
Stem LFD LAMP^b^	FIP/BIP, LF/LB, SF/SB	FITC	+	±	−	−	−	−	*40*	This study
LAMP^c^	F3/B3, LF/LB, FIP/BIP	N/A	+	±	−	−	−	−	*40*	This study
SAM-1 LAMP	F3/B3, LF/LB, FIP/BIP	N/A	+	+	+	−	−	−	*35*	[15]
Nested PCR test	F1/R1; F2/R2	N/A	+	+	+	−	−	−	*N/A*	[31]

^a^LAMP test with outer F3/B3 primers; ^b^LAMP test without outer F3/B3 primers. ^c^Primers designed in this study. ^d^The reaction done using 10^−4^ (1000 oocysts/ml). ^e^Time to recording a positive reaction. ^†^Approximately 30% of the replicates were consistently positive with preheated template and sequencing; N/A: not applicable.

**Table 3:** The comparative analysis of stem LFD LAMP, SAM-1 LAMP, and nested PCR test in the detection of previously confirmed and archived *C. hominis* and *C. parvum* DNA samples and clinical samples.

	Indices	Types of test		
		Stem LFD-LAMP	SAM-1 LAMP^a^	Nested PCR^b^
*Cryptosporidium* spp. DNA (*N* = 39)	Number of positive samples	29 (74.4%)	27 (69.2%)	25 (64.1%)

Clinical samples (*N* = 67)	Number of positive samples	16 (23.9%); 19 (28.4%)^c^	14 (20.8%)^†^	11 (16.4%)^†^
	Time to results (Min)^f^	80	120^d^	320^d^
	Accelerating primers	Loop and stem	Loop	nd

^a^SAM-1 LAMP test [15]; ^b^SSU rRNA nested PCR [31]. ^c^Template was preheated for 5 minutes. ^d^Detection using gel electrophoresis. ^f^From master mix preparation to visual result readout. ^†^The samples were also positive using stem LFD LAMP test; Nd: not done.

## Data Availability

The datasets supporting the conclusions of this article are included within the article.

## Abbreviations

LAMP: Loop-mediated isothermal amplification
FITC: Fluorescein isothiocyanate
LFD: Lateral flow dipstick
SAM-1: S-Adenosyl-methionine -1
HIV: Human Immunodeficiency Virus
FIP/BIP: Inner primer
LF/LB: Loop primers
SF/SB: Stem primers
GP 60: Glycoprotein 60
HSP 70: Heat shock proteins 70v.
